# Identification of the Key Molecular Drivers of Phosphorus Utilization Based on Host miRNA-mRNA and Gut Microbiome Interactions

**DOI:** 10.3390/ijms21082818

**Published:** 2020-04-17

**Authors:** Siriluck Ponsuksili, Henry Reyer, Frieder Hadlich, Frank Weber, Nares Trakooljul, Michael Oster, Puntita Siengdee, Eduard Muráni, Markus Rodehutscord, Amélia Camarinha-Silva, Jörn Bennewitz, Klaus Wimmers

**Affiliations:** 1 Leibniz Institute for Farm Animal Biology (FBN), 18196 Dummerstorf, Germany; reyer@fbn-dummerstorf.de (H.R.); hadlich@fbn-dummerstorf.de (F.H.); weber@fbn-dummerstorf.de (F.W.); trakooljul@fbn-dummerstorf.de (N.T.); oster@fbn-dummerstorf.de (M.O.); siengdee@fbn-dummerstorf.de (P.S.); murani@fbn-dummerstorf.de (E.M.); wimmers@fbn-dummerstorf.de (K.W.); 2 Institute of Animal Science, University of Hohenheim, 70599 Stuttgart, Germany; markus.rodehutscord@uni-hohenheim.de (M.R.); amelia.silva@uni-hohenheim.de (A.C.-S.); j.bennewitz@uni-hohenheim.de (J.B.); 3 Faculty of Agricultural and Environmental Sciences, University Rostock, 18059 Rostock, Germany

**Keywords:** phosphorus utilization, microRNA, mRNA, microbiota

## Abstract

Phosphorus is an essential mineral for all living organisms and a limited resource worldwide. Variation and heritability of phosphorus utilization (PU) traits were observed, indicating the general possibility of improvement. Molecular mechanisms of PU, including host and microbial effects, are still poorly understood. The most promising molecules that interact between the microbiome and host are microRNAs. Japanese quail representing extremes for PU were selected from an F2 population for miRNA profiling of the ileal tissue and subsequent association with mRNA and microbial data of the same animals. Sixty-nine differentially expressed miRNAs were found, including 21 novel and 48 known miRNAs. Combining miRNAs and mRNAs based on correlated expression and target prediction revealed enrichment of transcripts in functional pathways involved in phosphate or bone metabolism such as RAN, estrogen receptor and Wnt signaling, and immune pathways. Out of 55 genera of microbiota, seven were found to be differentially abundant between PU groups. The study reveals molecular interactions occurring in the gut of quail which represent extremes for PU including miRNA-16-5p, miR-142b-5p, miR-148a-3p, *CTDSP1*, *SMAD3*, *IGSF10*, Bacteroides, and Alistipes as key indicators due to their trait-dependent differential expression and occurrence as hub-members of the network of molecular drivers of PU.

## 1. Introduction

Phosphorus (P) is an essential mineral for all living organisms. Feed phosphates are produced from rock P stores that are a limited resource globally. On the other hand, the excessive use of P as fertilizer and feed supplement in agriculture has created environmental problems. The majority of P in animal feed comes from plant seeds. Up to 80% of P contained in plant seeds is in the form of inositol phosphates (InsPx), which cannot be efficiently utilized by monogastrics due to the lack or scarcity of endogenous phytase. Therefore, mineral feed phosphates are supplemented in diets of monogastric farm animals such as poultry and pigs. Measures to improve the utilization of InsPx and mineral P are needed to decrease the environmental load from animal production and preserve this valuable mineral resource.

In monogastric vertebrates, blood P homeostasis is maintained by tight regulation of enteral absorption, ostial mobilization, and renal excretion rates involving a number of known and yet to be elucidated regulators, transporters, endocrine, and paracrine signals. Phosphorus utilization (PU = P-accretion/P-intake) is a heritable trait. In broilers and laying hens, considerable heritability for P utilization was estimated [1,2]. In Japanese quail, a heritability of 0.14 was estimated for PU and quantitative trait loci (QTL) related to P utilization were identified [3]. A recent study showed that bone ash data are genetically correlated with PU and thus might be used as proxy traits to breed for an improved PU [4]. In pigs, genome-wide association study (GWAS) of inorganic phosphorus (IP) and alkaline phosphatase activity (ALP) in blood was reported and revealed candidate genes in the significantly associated genomic regions [5]. Furthermore, a remarkable change in the transcript level in response to P supply was identified in the porcine jejunum and kidney [6].

The absorption of mineral P and P from the cleavage of InsPx, which occurs mainly in the small intestine, is mediated by passive paracellular and active transcellular mechanisms. P absorption is driven by gut properties, microbiome composition, and interactions between the gut tissue and the microbiota. The interplay of host tissue function and microbiota composition can be obtained in a more detailed picture at molecular levels based on available high-throughput “omics” technologies. In particular, microRNAs (miRNAs) are promising candidates for regulatory interactions between the host and the gut microbiota due to their strong evolutionary conservation. Small non-coding RNAs, 21–25 nucleotides in size, are known as miRNA and are important for the regulation of gene expression. A recent study showed that fecal miRNA-mediated inter-species gene regulation facilitates host control of the gut microbiota [7]. Evidence of cross-kingdom regulation by miRNA was demonstrated by the ability of an exogenous plant-derived miR-168a to specifically downregulate the mammalian low-density lipoprotein receptor adapter protein 1 (LDLRAP1) mRNA and protein expression [8]. A wide range of dietary components such as amino acids, carbohydrates, fatty acids, and vitamins appear to affect expressions of miRNAs [9]. A few studies observed the relationship between diet and intestinal microbial activity. Broiler-fed diets differing in P, calcium (Ca), and phytase led to a shift in gut microbiota composition [10]. Furthermore, diets with high starch or high protein levels not only shifted the microbial composition but also changed miRNA expression [11]. In fact, molecular adaptations to low-P diets might contribute to the cleavage of P from InsPx and increase P absorption along the gastrointestinal tract [12].

MiRNAs also play important roles in bone metabolism, bone-related diseases, and bone cell development and function [13,14,15,16]. Previous studies demonstrated that mRNA–miRNA regulatory networks affect different phenotypes, including endometrial receptivity in cattle, divergent muscle properties such as muscle fiber type, metabolic enzyme activity, and ATP production both in vivo pigs and in vitro in cell culture [17,18,19]. In the context of PU, the miRNA(s) involved and the role of host miRNA–mRNA and microbiota interactions remain unclear.

Gut properties, the microbiome composition and interactions between the gut tissue and microbiota play a significant role in digestive capacity and need to be understood for improvement of PU. In this context, the objective of this study was to identify the gut miRNA and related mRNA targets associated with PU in the Japanese quail (Coturnix japonica). Phenotypically divergent Japanese quail representing extremes for the trait PU of an experimental population were selected for miRNA profiling of ileum tissue. Moreover, the mRNA expression and microbiota data were integrated with the miRNA readouts. Host mRNA–miRNA and microbiome regulatory networks in the ileum and their contribution to PU were characterized.

## 2. Results

Discordant quail pairs representing extremes (low vs. high) for the PU traits were selected from the 887 F2 quail population. In total, 21 quails with high P utilization (means ± SD (standard deviation) = 79.5 ± 3.5, *n* = 10) and low P utilization (means ± SD = 39.9 ± 11.1), *n* = 11) were selected for miRNA profiling of ileum tissue. Tissue samples of the ileum were collected and subjected to small RNA deep sequencing. Two outliers were excluded during data analysis based on the extremely low counts. The number of total reads was 110.4 Million of which 76.9 Million were clean reads. About 70% of the clean reads were mapped to the *C. japonica* 2.0 genome—Genome (https://www.ncbi.nlm.nih.gov/assembly/GCF_001577835.1/). The number of total reads, clean reads, and mapped reads obtained from sequencing are listed in Table 1 for all 19 libraries. About 49.6% of the clean reads were mapped to miRNA by using miRDeep2.

### 2.1. Differential Expression Analysis between PU Groups

**Figure 1:**
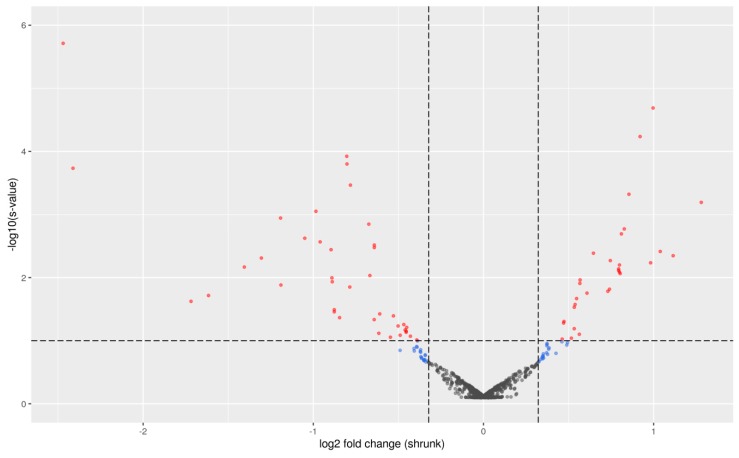
Differential miRNA expression for the phosphorus utilization (PU) group (“high” vs. “low” *n* = 19): Volcano plot showing $-\log_{10}\left(s\right)$ (with s denoting the *s*-values) versus the shrunken log2 fold changes (LFCs; calculated using the “apeglm” method in the function IfcShrink() from R package DESeq2). Red dots denote miRNAs having an *s*-value below the threshold of 0.10 and an absolute shrunken LFC above the threshold of $\log_{2}\left(1.25\right)\;\approx 0.322$. Blue dots denote miRNAs having an *s*-value above the threshold of 0.10 and an absolute shrunken LFC above the threshold of $\log_{2}\left(1.25\right)\;\approx 0.322$. Gray dots denote miRNAs having an *s*-value above the threshold of 0.10 and an absolute shrunken LFC below the threshold of $\log_{2}\left(1.25\right)\;\approx 0.322$. Dashed lines also show the thresholds.

In total, 1118 miRNAs sequences were used for downstream analyses. miRDeep2 was used to identify novel miRNA with chicken as the reference species since there was no miRNA reference information available for the quail. Our data showed the presence of 509 novel miRNA and 609 miRNAs conserved between chickens, mice, or humans. Figure 1 shows a volcano plot of the results of all 1118 miRNAs.

**Figure 2:**
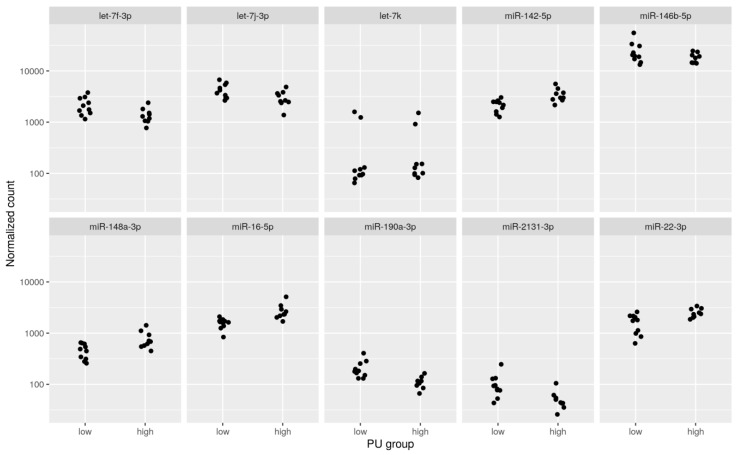
Normalized counts of miRNA expression pattern between P utilizations groups (PU) for the top 10 known miRNAs with the lowest *s*-values between PU groups (*n* = 19). The plot was created using the DESeq2 function plotCounts() and the R package ggplot2.

This volcano plot illustrates the association of 69 miRNAs with the PU group (red dots) at *s* < 0.10. Of these, 21 out of 69 were novel miRNA and 48 were found to be conserved in humans, mice, or chickens (Table S1). The top 10 significantly different known miRNA between PU groups were miR-22, miR-148a-3p, miR-146b-5p, let-7f-3p, miR-16, let-7j-3p, miR-2131-3p, miR-142-5p, let-7k, and miR-190a-3p. Plots of the normalized read counts are shown in Figure 2 for the top 10 known miRNAs with the lowest *s*-values between PU groups. The highest *s*-value among the 10 miRNAs was approximately 0.004. Those plots confirm that the ranking of the miRNAs by *s*-value is meaningful as most of the miRNAs show differential expression patterns between the “low” and the “high” PU group.

### 2.2. Correlation between miRNA and mRNA

**Figure 3:**
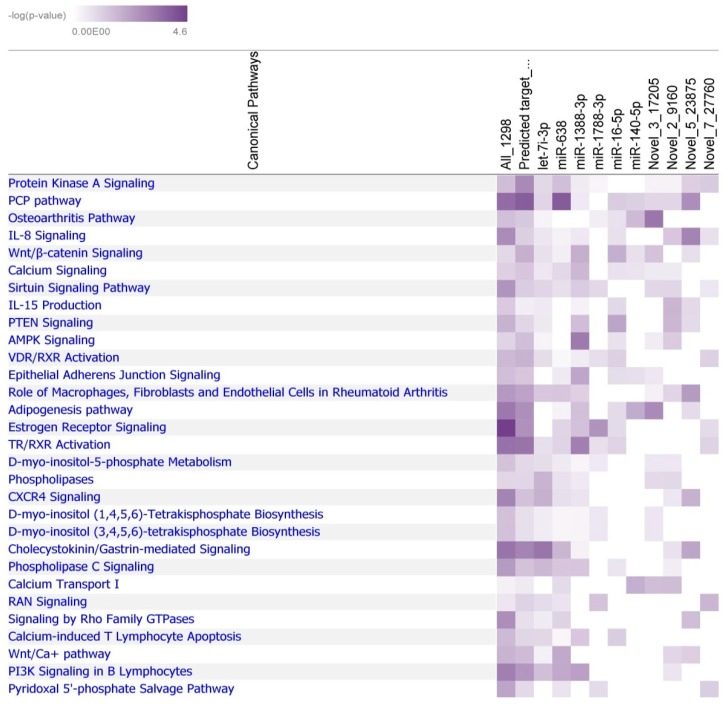
Heatmap showing enriched canonical pathways derived from analyses of miRNAs and corresponding mRNAs based on (1) negative correlation of expression in the same samples (All_1298), (2) negatively correlated expression and target prediction, and (3) negative correlated and predicted target transcripts of let-7i-3p, miR-638, miR-1388-3p, miR-1788-3p, miR-16-5p, miR-140-5p, novel-3-17205, novel-2-9160, novel-5-23875, and novel-7-27760. The intensity of color indicates significance from light to dark.

The mRNA expression data of the same samples from our previous study [20] were used for pairwise correlation analysis. The threshold for differential expression was set at an *s*-value of 0.10 for miRNA and a *q*-value of 0.10 for mRNA for the pairwise correlation analysis. In total, 4378 pairs with a negative correlation between miRNA (70 miR sequences) and mRNA (1298 genes) were identified. Negative correlations between differentially expressed miRNA and mRNA ranged from |0.82| to |0.45| (*p* < 0.05). A highly significant negative correlation pair identified in the context of PU was of miR-146b-5p and *PLS3* (*r* = −0.77, *p* < 0.0001). The negatively correlated genes were subjected to functional analysis and found to be highly enriched in (Figure 3).

### 2.3. Prediction of miRNA Targets in Japanese Quail

Japanese quail genome version “Coturnix_japonica 2.0” was used for predicting miRNA targets. Functional network analysis was done to gain biological insights into their predicted targets. After combining the correlation analysis and target prediction results, 945 miRNA-mRNA pairs containing 609 genes and 54 mature miRNA sequences were retained. All 609 genes were enriched in the PCP pathway, TR/RXR activation, and cholecystokinin/gastrin-mediated signaling. Some miRNAs like NW_015440444.1_47932_mature (corresponding to ppy-miR-638 with two mismatches) and let-7i-3p had multiple targets, 281 and 183 genes, respectively. About 10 miRNAs were predicted to target more than 20 genes and these were subjected to functional analysis. The significant canonical pathways (*p* < 0.01) are shown in Figure 3. The predicted target transcripts of miR-638, miR-1388, novel miRNA 5_23875, and miR-16-5p belong to the PCP, Wnt/Ca+, and Wnt/β-catenin signaling pathways including the genes *ATF2, LGR4, PRICKLE1, ROCK1, RSPO3, WNT5A*, *NFAT5*, and *PLCB1*. Other pathways identified were related to osteoarthritis, RAN signaling, estrogen receptor signaling, D-myo-inositol-5-phosphate metabolism, or pyridoxal 5’-phosphate salvage pathway. Interestingly, the genes in these pathways are the targets of many miRNA including miR-1788-5p, miR-140-5p, let-7i-3p, or novel miRNA 3_17250. *INPP5F* and *ITPK1*, which belong to 1D-myo-inositol hexakisphosphate biosynthesis II, were predicted to be targets of let-7i-3p. *ADAMTS5, NOTCH1, SMAD9*, and *SOX9* genes, enriched in the osteoarthritis pathway, were predicted as targets of a novel miRNA (3_17250). In addition, immune pathways like IL-8 Signaling, IL-15 production, and P13K Signaling in B Lymphocytes were also identified from correlated and predicted targets.

### 2.4. Differences in Gut Microbiota Composition among PU Group

**Figure 4:**
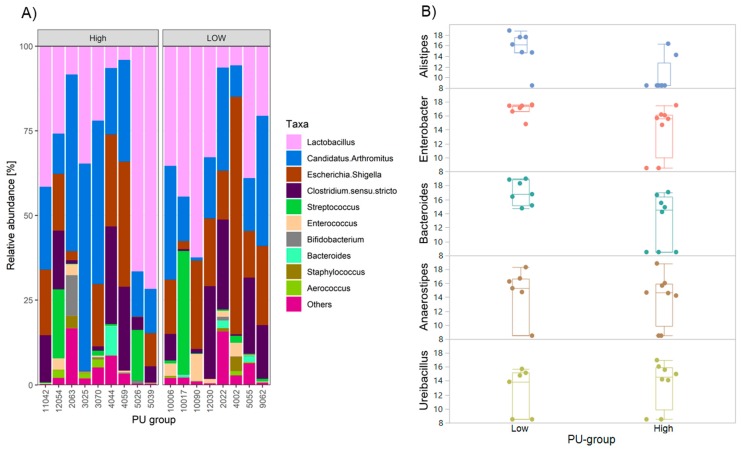
Characterization of microbial community composition and phosphorus utilization (PU; *n* = 17). (**A**) Microbiota variation at the genus level of ileum digesta samples of Japanese quail with high and low PU. Displayed is the relative abundances of the 10 most prevalent genera. (**B**). Significantly different (*q* < 0.05) genera between high and low phosphorus utilization samples. *X*-axis indicates the normalized operational taxonomic units (OTU) using DESeq2. Each point represents a normalized OTU.

The microbiota data from ileum digesta samples were used. Operational taxonomic units (OTUs) were grouped at the genus level and very low occurring taxa were removed from the microbial data set. Following this, 55 genera were further included in the analysis. The taxonomic characterization of the microbiota including the top 10 genera is shown for all individual quail samples in Figure 4A. The most abundant genera were *Lactobacillus* and *Candidatus arthromitus*. Seven out of 55 genera were found to be differentially abundant between PU groups at *q* < 0.05. These include *Alistipes*, *Enterobacter*, *Bacteroides*, *Anerostipes*, *Ureibacillus*, *Tepidimicrobium*, and *Planifilum*. The differences between PU groups of the top five of these genera are shown in Figure 4B.

### 2.5. Identification of the Molecular Drivers of Host–Gut Microbiome-Based PU

**Figure 5:**
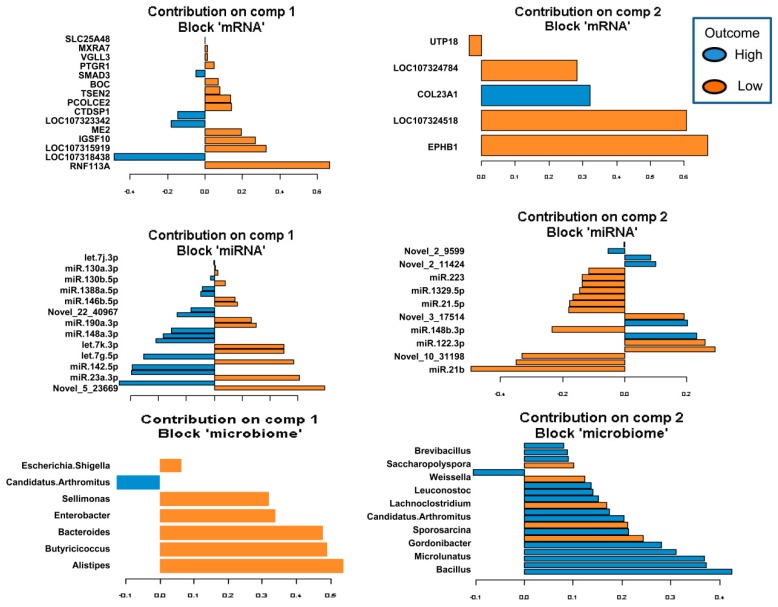
The optimal omics biomarker set of host mRNA, miRNA and microbial genera which correlated with the phosphorus utilization (PU) groups low and high over 2 component sets. The minimal set of features selected by DIABLO across data types could discriminate between high and low PU groups in DIABLO Components 1 and 2. Blue indicates a high PU group and orange indicates a low PU group. (DIABLO: Data Integration Analysis for Biomarker discovery using Latent cOmponents).

**Figure 6:**
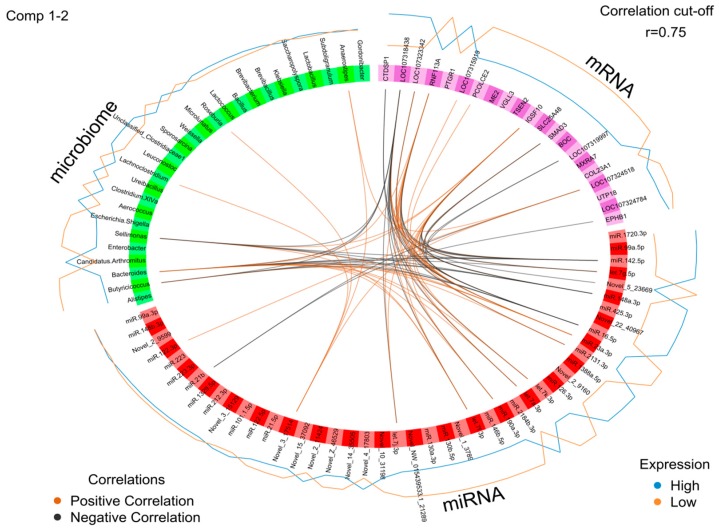
Variable plot of miRNA, mRNA, and microbiota with a link to phosphorus utilization (PU) groups. The optimal omics bio-signature consisted of 21 mRNA, 45 miRNA, and 27 microbial genera over two component sets. Brown connections indicate a positive correlation, while black connections indicate a negative correlation between the mRNA, miRNA, or microbiota. Blue and orange lines in the outer circle indicate the level of expression in either the high or the low PU group (*n* = 15).

**Figure 7:**
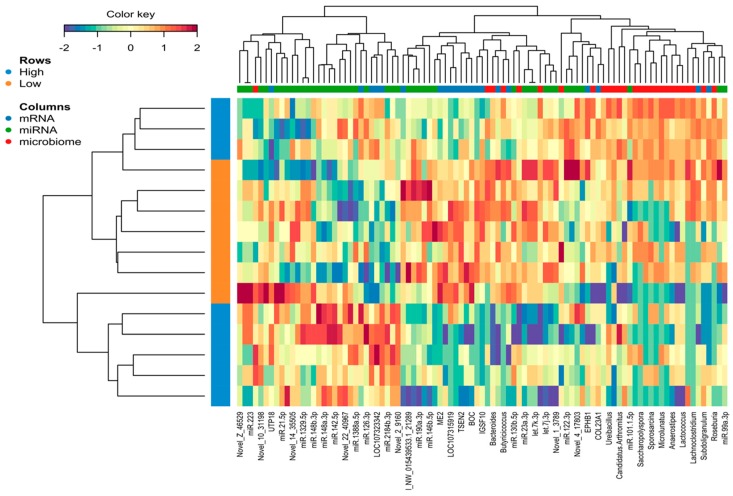
Heatmap of expression values of all variables (mRNA, miRNA, and microbiota) that are part of the multi-omics biomarker panel. Expression values linked with phosphorus utilization (PU) were scaled and underwent hierarchical clustering. The color key shows the correlation levels of the heat map. The vertical cluster shows samples with high PU and low PU (*n* = 15). The horizontal cluster shows the multi-omics biomarker panel.

**Figure 8:**
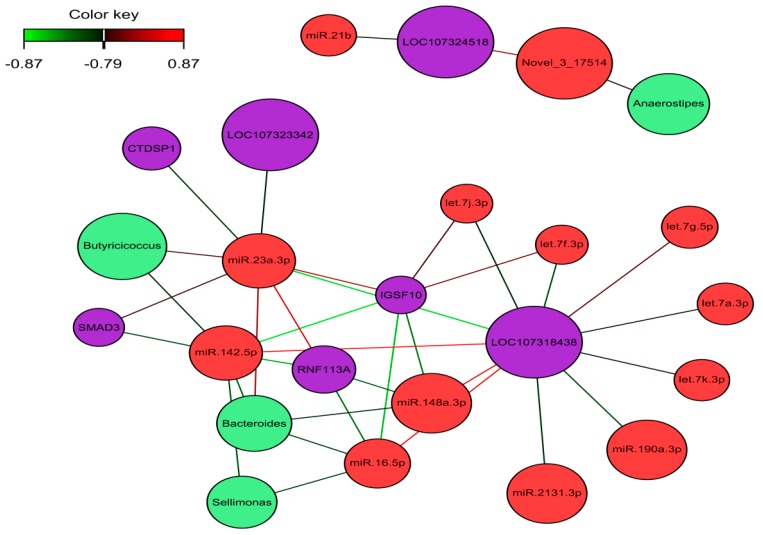
Network visualization of miRNA, mRNA, and microbiota panel, highlighting correlated variables (*r* > |0.79|). Color nodes indicate microbes in green, mRNA in violet and miRNA in red. Edge color indicates the correlation between nodes as shown in the color key. Green edges indicate a negative correlation, red edges indicate a positive correlation.

The omics datasets (miRNA, mRNA, and microbiota) measured on the same samples were normalized and filtered. In total, 12,428 transcripts, 926 miRNA, and 55 microbial genera from the same samples were used as input for the analysis of a host–gut biomarker panel linked to PU. DIABLO is a multivariate approach used to integrating a complex dataset with a small number of samples and heterogeneous data. The software constructs components, i.e., linear combinations of miRNA, mRNA, and microbiota, which are maximally correlated across all input data types with a specific outcome variable (in this case high and low PU group). Minimal marker selection associated with the outcome groups is simultaneously performed [21]. The optimal omics bio-signature was identified in this study consisting of 21 mRNA, 45 miRNA, and 27 microbial genera over two component sets. The contribution to Components 1 and 2 of block mRNA, miRNA, and microbiome is shown in Figure 5). Interestingly, in the microbiota block1, only *Candidatus arthromitus* was positively associated with the high PU group. A CircosPlot demonstrating the correlation among different omics block is shown (Figure 6, correlation cutoff: *r* > |0.75|). The heat map of the gut biomarker panel showed that the “low” PU group clustered together whereas the “high” PU group was distinct (Figure 7). The network of top molecules with correlation levels greater than 0.8 is demonstrated in Figure 8. Five miRNAs in the let7 family including let-7j-3p, let-7g-5p, let-7k-3p, let-7a-3p and let-7f-3p belonged to the top biomarkers panel for PU. In addition, miRNAs, which were also found among the top differentially expressed miRNAs between PU groups, such as miR-2131-3p and miR-190a-3p as well as miR-142-5p, miR-148a-3p, miR-16-5p and miR-23a-3p were elements of the network with the latter four being highly connected hubs within the network of panel elements (Figure 8). Microbial variables like *Sellimonas*, *Butyricicoccus*, *Bacteroides*, *Alistipes*, and *Anaerostipes* were identified in this panel. Transcripts including *RNF113A, LOC107318438* (zinc finger protein 664-like), *IGSF10*, *LOC107323342* (neuroblastoma suppressor of tumorigenicity 1, NBL1), *CTDSP1*, *SMAD3*, and *LOC107324518* (C-reactive protein-like) were strong biomarkers linked to PU groups.

## 3. Discussion

Phenotypic variation and heritability of PU were observed in the broiler, laying hens, and Japanese quail [1,2,3]. Complex traits such as PU result from the interplay of genetic or non-genetic factors and effects of microbial communities in the gut. Our aim was to identify novel molecular routes affecting PU by detecting differentially expressed miRNAs in the ileum of PU divergent Japanese quail. Gut miRNAs regulate mRNA expression of the host intestine and may shape the gut microbiota. Therefore, mRNA expression and microbiome data were correlated with the miRNA expression data in this study.

Change in dietary P prompts an altered gene expression in the intestine and kidney and also led to a shift in gut microbiota composition [10,22]. In addition, a low P diet induced negative effects on feed intake and body weight gain [23]. Taken together, P change in the diet leads not only to the phenotype change but also to shifts at the molecular level. In this study, the birds were fed with a low-P diet in order to stimulate their full genetic potential of PU. Extremes for PU were selected out of an F2 population for miRNA profiling of the ileal tissue. Accordingly, we found that low PU was associated with lower body weight gain and changes at the molecular level including miRNAs, mRNA targets, and gut microbiota. Finally, a number of biomarker panels associated with the PU were provided, which may have the potential to improve the PU by breeding.

Many factors play a significant role in the regulation of P homeostasis including parathyroid hormone, vitamin D, calcitonin, and calcium metabolism. Differential expressions of miRNA or its targets between PU groups were enriched in functional pathways involved in phosphate or bone metabolism such as RAN, estrogen receptor, and Wnt signaling.

The regulation of bone metabolism and Wnt signaling is well studied [24,25]. A number of molecular pathways respond to the modulation of P supply and are related to processes affecting bone mineral density and microarchitecture [26,27]. Enrichment analysis pointed out immune pathways like IL-8 Signaling, IL-15 production, and P13K Signaling in B Lymphocytes. This is in line with our previous study that showed a close association between dietary P supply and transcripts enriched in the immune system [23]. The current study shows the role of miRNAs in linking transcripts enriched in bone metabolism and immune functions. Many miRNAs are known to regulate bone metabolism, host-microbiome interaction, and inflammatory and immune processes [16,28,29]. MiR-146a affects osteoblast and osteoclast formation in vitro and in vivo miR-146a+/− mice [16,30]. Using computational analysis and an in vitro cell culture system, miR-146b levels were shown to be significantly associated with chronic kidney disease and mineral bone disorder [31]. MiR-146a was reported as a key molecule in the interaction between intestinal epithelial cells, microbial components, and inflammatory stimuli [29]. In our study, miR-146b, one of the top 10 differentially expressed miRNAs between PU groups was found to show a significant negative correlation with *PLS3*, *CST7*, *GAL3ST1*, *ENPP6*, *ALOX5AP*, and *CD8A*. These differentially expressed genes between PU groups were associated with bone metabolism or immune responses. For instance, mutations of the gene encoding plastin-3 (*PLS3*) were associated with severe primary osteoporosis [32,33] and shown to affect bone mineral homeostasis through regulation of osteoclast activity [34]. *CST7* was upregulated by the induction of *Runx2*, an osteoblast master transcription factor, in C4-2B cells [35]. *ENPP6* activities mediate bone mineralization which is a key process in the formation of bone [36]. *ALOX5AP* (encoding leukotriene-synthesis enzymes) and *CD8A* (encoding the T-cell surface glycoprotein CD8 alpha chain) are both involved in inflammatory and immune responses [37].

MiR-148a, another differentially expressed miRNA between PU groups, is a well-known miRNA associated with osteoporotic osteoclasts [38], affecting osteogenic differentiation [39], and involved in bone remodeling [40] and bone homeostasis and metabolism [41]. Another top differentially expressed miRNA between PU groups was miR-142-5p, which is known to induce osteoblastogenesis during the bone healing process [15] and regulate inflammation by influencing T cell differentiation [42]. It is also associated with gut diseases [43,44]. Jun dimerization protein 2 (*JDP2*) is negatively correlated with three novel quail miRNAs (8_28985_mature, 2_12370_mature, and Z_45830_mature), which were highly differentially expressed between PU groups. Interestingly, *JDP2* is a critical regulator in bone mineral homeostasis and osteoclastogenesis [45].

The relationship between microbiota composition and bone metabolism has been reported [46,47]. Attempts have been made to explain how the microbiome can affect bone homeostasis either though the immune system’s response [48] or influence on hormone levels such as parathyroid hormone (PTH) or vitamin D metabolites [49]. Recent evidence suggested communication between the gut microbiome and host can take place via miRNA which is conserved between species and can regulate transcripts across species [7,50]. In this study, the integration analysis of mRNA, miRNA, and microbiota provided molecular drivers of host–gut microbiome under different PU groups. The deduced molecule panels showed the possible interactions occurring in the gut of animals which represent extremes for P utilization. Interestingly, microbes like *Alistipes, Anaerostipes, Enterobacter* and *Bacteroides* which were differentially regulated between PU groups were also identified in the omics panel. *Bacteroides, Butyricicoccus*, and *Sellimonas* were highly correlated with miR-142-5p, miR-16b-5p, miR-23a, and miR-148-3p, and the transcripts of *RNF113A, IGSF10, LOC107323342* (neuroblastoma suppressor of tumorigenicity 1(*NBL1*)), *CTDSP*1, and *SMAD3*. These miRNAs are involved with bone metabolism or as key molecules in the interaction among intestinal epithelial cells, microbial components, and inflammatory stimuli [29]. MiR-23a belongs to this molecular panel and links LOC107323342 (neuroblastoma suppressor of tumorigenicity 1, NBL1) and *Butyricicoccus*. Suppression of miR-23a-3p promoted osteoblast proliferation and differentiation, and alkaline phosphatase activity by targeting the PGC-1α/WNT/β-catenin signaling pathway in osteoporotic rats [51]. Bone morphogenetic proteins (BMPs) which play an important role in post-natal bone formation were antagonized through the action of numerous extracellular proteins, including NBL1 [52], SMAD2/3, and CTDSP1/2 [53]. *Butyricicoccus,* a butyrate-producing bacterium belonging to the class Clostridia, is decreased in the high PU quail. *Butyricicoccus* is a mucosa-associated bacterial genus reported to be under-represented in the colonic mucosa of patients with active ulcerative colitis [54]. Five miRNAs of the let-7 family belong to the molecular panel for PU. Previous studies found that the expression of the let-7 family correlated significantly with glucose levels and regulates glucose metabolism [55,56]. *IGSF10* was negatively correlated and predicted as a target of miR-148a-3p and miR-142a-5p in this study. This molecule was found again in the panel and direct and directly linked to *Butyricicoccus, Bacteroides*, and *Sellimonas*.

In conclusion, differentially expressed miRNAs were found in the ileum of Japanese quail with high or low PU. The miRNAs identified including miR-148a-3p, miR-146b-5p, miR-142-5p, miR-16-5p, and miR-23a, were previously reported to be involved in bone metabolism, immune system regulation, and modulating the microbiome. Negative correlation and target prediction of differentially expressed miRNAs and mRNAs in Japanese quail revealed enriched pathways including Wnt signaling, RAN signaling, and estrogen receptor signaling that relates to P metabolism. Indeed, pathways and signaling events between tissues attributed to mineral homeostasis in PU. Integration of host omics and gut microbiome data provided a list of molecular drivers that influence PU in Japanese quail. Our study of Japanese quail gut microbiota, mRNA and miRNA shows molecular interactions occurring in the gut of quail with high or low PU. In particular, the study reveals miRNA-16-5p, miR-142b-5p, and miR-148a-3p as key indicators of PU due to their trait-dependent differential expression and occurrence as hub-members of the network of molecular drivers of PU.

## 4. Materials and Methods

### 4.1. Experimental Design and Samples Selection

The F2 cross of Japanese quail (*Coturnix japonica*) used in this study originated from a previous study [3,4]. The experiment was conducted in accordance with the German Animal Welfare Legislation approved by the Animal Welfare Commissioner of the University of Hohenheim. An F2-design using two Japanese quail lines divergently selected on social reinstatement behavior was established with 12 males from Line A and 12 females from Line B of the F0-generation. A total of 17 roosters and 34 hens were randomly selected from the F1-birds to generate F2-animals [3]. Quail hatchlings were raised in groups in floor pens on wood shavings until they were transferred to metabolic boxes and kept individually when they were 8 days old. The floor of the boxes was covered with a P-free filter paper to facilitate excreta collection. The room temperature was adjusted to 35 °C at the day of placement and gradually reduced to 25 °C on day 15 of age. Housing conditions and feed composition were explained in more detail by Beck et al. [3]. In order to let the birds express their full genetic potential of PU, the F2-animals were fed with a low-P diet (4.0 g/kg DM) without a mineral P supplement or phytase. PU was calculated from the ratio between total P intake and P excretion for each individual [3].

The discordant quail sib pair representing extremes (low vs. high) for the PU traits was selected from the 887 F2 quail population. Animals from 10 families with significant differences in PU were selected. In addition, the same sexes of birds in the PU groups of the family were considered. In total 21 quails with high PU and low PU were selected for miRNA profiling of ileum tissue. After filtering outlier animals in terms of their miRNA expression, 19 quails still remained.

### 4.2. RNA Extraction

Japanese quail were humanely sacrificed to collect tissue samples. For this experiment, a 1.5 cm long section of the intestine was dissected out of the ileum, cut open, and rinsed with a sterilized saline buffer to remove digesta residue. The whole tissue samples were immediately submerged in a solution of RNAlater (Sigma-Aldrich, Taufkirchen, Germany) and stored at −80 °C until RNA extraction. Total RNA was extracted from approximately 50 mg of the sample using TRIzol Reagent (Invitrogen) and the RNeasy Mini kit (Qiagen, Hilden, Germany) and further enriched for small RNA fractions using the miRNeasy Mini kit (Qiagen, Hilden, Germany). The integrity of total RNA was assessed using an Agilent RNA 600 Nano kit and the enrichment and concentration of miRNAs were determined using the Agilent Small RNA kit and the 2100 Bioanalyzer system (Agilent Technologies, Waldbronn, Germany).

### 4.3. Small RNA Library Preparation and Sequencing

Small RNA sequencing libraries were generated from 1 µg enriched small RNA using the SMARTer smRNA-Seq Kit for Illumina (Takara Bio Europe SAS, Saint-Germain-en-Laye, France). Essentially, small RNAs were polyadenylated at the 3′-end to provide a priming site for the 3′smRNA dT primer and reverse transcribed into first-strand cDNA using the Moloney murine leukemia virus (MMLV)-derived PrimeScript reverse transcriptase (RT). The SMART smRNA oligo was added to the 5-end via the locked nucleic acid (LNA) technology. Illumina indexing adapters were incorporated during an additional PCR amplification to enable sample multiplexing. The smRNA-seq libraries were quality assessed for the expected fragment length (major peak at about 175 bp) using the Agilent High Sensitivity DNA kit and the 2100 Bioanalyzer (Agilent Technology, Waldbronn, Germany). Size-selection was performed using the BluePippin System and 3% agarose gel cassettes with an internal Q2 DNA marker and size-selection parameters of 148–185 bp (Sage Science, Beverly, MA, USA). The molar concentration of the libraries was determined using the Qubit dsDNA HS assay kit (Invitrogen, Darmstadt, Germany). Sequencing reads were cluster-generated using the cBot system and sequenced for 50 bp single-end reads on the HiSeq2500 sequencing platform at the Institute of Genome Biology, FBN Dummerstorf, Germany. The base call (BCL) files from the sequencing run were de-multiplexed and converted into the FASTQ files using the bcl2fastq2 conversion software, v. 2.19 (Illumina, San Diego, CA, USA). The raw fastq files were quality-checked using FastQC, version 0.11.5. The raw data was submitted to a public database, ArrayExpress with the accession number E-MTAB-8587.

### 4.4. Pre-Processing—Adapter Trimming, Quality Control, and Read Collapsing

Short RNA library raw reads were obtained from the ileum tissue samples using Illumina HiSeq sequencing. In the first step, adapter trimming and quality control were applied using flexbar and fastqc to filter out contaminated sequences.

We identified and quantified known and novel miRNAs using miRDeep2 [57]. The chicken was used as the reference species since there are no known miRNAs for the quail. Humans and mice were used as related species. Since the resulting read count matrix included multiple rows with the same miRNA identifier, but different mature miRNA sequences, the mature miRNA sequences were used to identify the miRNAs (instead of their identifiers given by miRDeep2). Afterwards, there were still duplicated miRNAs (now identified by their mature sequence) in the read count matrix. To solve this issue, the maximum number of reads per mature miRNA sequence was taken as the read count for the subsequent analyses. This was done for each sample separately. The result showed 5963 miRNAs among which 526 were novel. As a pre-filtering step, we only kept miRNAs where the 75% quantile of the log counts per million (log CPM) transformed read counts was greater than $-\log_{2}\left(L_{min}*10^{-6}\right)$ with $L_{min}=2\;686\;672$ denoting the minimum library size in our data, i.e., $-\log_{2}\left(L_{min}*10^{-6}\right)\;\approx -1.426$. The log CPM values were calculated using the function cpm() from the R package edgeR [58] with a prior count of 1. The number of miRNAs kept after applying this filtering rule was 1118. These 1118 miRNAs were used for downstream analyses.

### 4.5. Data Analysis miRNA

Before analyzing the read count data, the two outlier samples with outstanding low counts (more than 2 SD lower) were removed and their families pooled to yield a common family. Since the data still exhibited batch effects even after adjusting for family, PU group, and sequencing lane, we used the function RUVr() from the R package RUVSeq [59] to estimate batch variables (BVs). The function RUVr() required the calculation of residuals from the model fit with all desired predictors, in our case, “family”, “PU group”, and “sequencing lane”. We followed the approach given in RUVSeq’s vignette (package version: 1.18.0) for calculating the corresponding deviance residuals. After estimating all possible BVs using RUVr(), we decided to include the first 2 BVs (BV1 and BV2) in the final model.

The R package DESeq2 [60] was used for the differential expression analysis. The following predictors were used in the DESeq2 model: family, PU group (“high” versus “low”), sequencing lane (BV1 and BV2). The default settings of DESeq2 function DESeq() were used. In order to decide for interesting miRNAs (i.e., those that are differentially for the PU group), we calculated *s*-values using the DESeq2 function lfcShrink() with the “apeglm” method [61]. The “independent filtering” that is by default applied by DESeq2 was turned off. We used *s*-values instead of *q*-values since the *p*-value distribution showed an outstanding peak at high *p*-values, violating the assumption of uniformly distributed *p*-values under the null hypothesis that is necessary for *q*-values. Since we supplied a threshold for the log2 fold change (LFC) of $\log_{2}\left(1.25\right)\;\approx 0.322$, the *s*-value represents here not the *false sign rate*, but the *false sign or small rate,* where “small” denotes an LFC in the interval $\left[-\log_{2}\left(1.25\right),\;\log_{2}\left(1.25\right)\right]$. Thus, the *s*-value of a given miRNA is the average error probability (more precisely: the average local false sign or small rate) among the miRNAs with lower or equal *s*-value which is, in our opinion, quite an intuitive interpretation compared to quantities based on *p*-values.

### 4.6. Prediction of miRNA Targets and Correlation between miRNA and mRNA Profiles in Japanese Quail

Based on ensEMBL annotation version 97, 5106 3’UTR sequences, 5662 5’UTR sequences, and 15,732 coding sequences were extracted from the Japanese quail (*C_japonica*) genome. Next, these sequences were split into 2 kb fragments with a 50-base overlap. Finally, the outputs were investigated as being potential linkage targets to the given miRNA using RNAhybrid version 2.1.2 with binding energy cutoff –25 k, helix constraint 2 to 7, and one hit per target. Each potentially hybridizing miRNA-mRNA pairing is summarized by its minimum free energy and its *p*-value.

DESeq2 was used for calculating variance-stabilizing transformations of the miRNA and the mRNA count matrices derived from the same animals in a complementary study [20]. Afterwards, the function removeBatchEffect() from the R package limma [62] was used to remove the batch effects of sequencing lane, BV1, and BV2 from the transformed miRNA expression matrix. For the transformed mRNA expression matrix, the 2 samples that were outlier samples for the miRNA data were removed. Finally, Pearson correlations between miRNA and mRNA profiles (access number E-MTAB 8587) were calculated.

In order to identify the functional potential of miRNA target genes, IPA software (Ingenuity System, https://www.ingenuity.com) was used. It categorizes genes based on annotated gene functions and statistically tests for over-representation of functional terms within the gene list using Fisher’s Exact Test (*p* < 0.05).

### 4.7. Microbiome Data Analysis

Operational taxonomic units (OTUs) deduced from 16S rRNA sequencing of the same animals were obtained from a recent study (access number PREJB37544) [63]. Initially, OTUs were assigned to taxa at the genus level and OTU counts belonging to the same genera were summarized. Moreover, the dataset was filtered so that only taxa with more than one observation in at least half of the samples were considered. To identify differentially abundant taxa between high and low PU groups, data were analyzed at the genus level using the DESeq2 package in the R environment [60]. Therefore, a negative binominal Wald test was applied considering family and PU group in the statistical model. Genera that differed between PU groups with *q* < 0.05 were considered significant.

### 4.8. Data Integration of the Microbiota, mRNA, and miRNA

The normalized OTU abundances of microbiota, mRNA read counts, and miRNA read counts were transformed using a variance-stabilizing transformation method implemented in DESeq2 and used as input for further analysis. In order to identify a highly correlated multi-omics signature discriminating between PU groups, the multi-block discriminant analysis with DIABLO (Data Integration Analysis for Biomarker discovery using Latent cOmponents) embedded in R package “mixOmics” (version 6.6.2) [21,64] was used. Host mRNA, miRNA, and microbial data were used as input for identifying the molecular drivers for Japanese quail PU.

## Figures and Tables

**Table 1:** Read counts in million (M) and mapping statistics, obtained from all samples.

Probe	Family	Sex	P-Utilization	Groups	Total Reads Counts (M)	Mapped Reads (M)	Unmapped Reads (M)	% Mapped	% Unmapped
2063	1	Female	79.67	High	4,643,858	3,152,958	1,490,900	67.90	32.11
4002	1	Female	50.81	Low	3,905,920	3,002,397	903,523	76.87	23.13
5039	2	Female	76.20	High	7,514,630	4,940,662	2,573,968	65.75	34.25
10,006	2	Female	43.06	Low	6,148,717	4,457,473	1,691,244	72.49	27.51
5026	3	Female	83.43	High	6,311,029	3,986,110	2,324,919	63.16	36.84
7023	3	Female	52.59	Low	3,977,952	2,849,897	1,128,055	71.64	28.36
3025	4	Male	79.16	High	4,625,821	3,029,840	1,595,981	65.50	34.50
10,017	4	Male	39.75	Low	4,229,818	2,847,346	1,382,472	67.32	32.69
5055	4	Male	44.71	Low	5,273,807	4,082,562	1,191,245	77.41	22.59
11,042	5	Male	79.00	High	7,237,297	4,880,845	2,356,452	67.44	32.56
3070	6	Male	86.77	High	9,339,712	6,706,669	2,633,043	71.81	28.19
12,030	6	Male	21.49	Low	4,751,504	3,588,653	1,162,851	75.53	24.47
12,054	7	Male	77.83	High	5,903,443	3,990,231	1,913,212	67.60	32.41
6039	7	Male	27.77	Low	3,881,743	2,581,642	1,300,101	66.51	33.50
4059	8	Female	77.02	High	9,276,166	6,402,170	2,873,996	69.02	30.99
2022	8	Female	45.65	Low	5,061,335	3,524,773	1,536,562	69.64	30.36
10,090	9	Female	48.29	Low	4,239,513	2,835,088	1,404,425	66.87	33.13
8017	10	Male	76.26	High	7,515,392	5,416,718	2,098,674	72.08	27.93
6035	10	Male	24.93	Low	5,011,594	3,633,621	1,377,973	72.50	27.49

## Supplementary Materials

Supplementary materials can be found at https://www.mdpi.com/1422-0067/21/8/2818/s1. Table S1. A list of differentially expressed miRNA derived from ileal tissue of quails discordant for phosphorus utilization. Associated statistics (*p*-value, *s*-value, log2Fold change) are provided.

## Abbreviations

OTUs	Operational taxonomic units
P	Phosphorus
PU	Phosphorus utilization
OTUs	Operational taxonomic units
miRNA	microRNA
